# Case report: Glycaemic management and pregnancy outcomes in a woman with an insulin receptor mutation, p.Met1180Lys

**DOI:** 10.1186/s40842-024-00166-9

**Published:** 2024-03-10

**Authors:** Mairéad T. Crowley, Eirena Goulden, Begona Sanchez-Lechuga, Aileen Fleming, Maria Kennelly, Ciara McDonnell, Maria M. Byrne

**Affiliations:** 1 Department of Diabetes and Endocrinology, Mater Misericordiae University Hospital, Dublin, 7 Ireland; 2 Rotunda Maternity Hospital, Dublin, Ireland; 3 Department of Paediatric Endocrinology & Diabetes, CHI at Temple Street, Dublin, Ireland

**Keywords:** Insulin receptor mutation, Type A insulin resistance, Diabetes in pregnancy, Monogenic diabetes, Hyperinsulinaemic hypoglycaemia

## Abstract

**Background:**

Heterozygous insulin receptor mutations (INSR) are associated with insulin resistance, hyperglycaemia and hyperinsulinaemic hypoglycaemia in addition to hyperandrogenism and oligomenorrhoea in women. Numerous autosomal dominant heterozygous mutations involving the INSR β-subunit tyrosine kinase domain resulting in type A insulin resistance have been previously described. We describe the phenotype, obstetric management and neonatal outcomes in a woman with type A insulin resistance caused by a mutation in the β-subunit of the INSR.

**Case presentation:**

We describe a woman with a p.Met1180Lys mutation who presents with hirsutism, oligomenorrhoea and diabetes at age 20. She has autoimmune thyroid disease, Coeliac disease and positive GAD antibodies. She is overweight with no features of acanthosis nigricans and is treated with metformin. She had 11 pregnancies treated with insulin monotherapy (*n* = 2) or combined metformin and insulin therapy (*n* = 9). The maximum insulin dose requirement was 134 units/day or 1.68 units/kg/day late in the second pregnancy. Mean birthweight was on the 37th centile in INSR positive offspring (*n* = 3) and the 94th centile in INSR negative offspring (*n* = 1).

**Conclusion:**

The p.Met1180Lys mutation results in a phenotype of diabetes, hirsutism and oligomenorrhoea. This woman had co-existent autoimmune disease. Her insulin dose requirements during pregnancy were similar to doses observed in women with type 2 diabetes. Metformin may be used to improve insulin sensitivity in women with this mutation. Offspring inheriting the mutation tended to be smaller for gestational age.

## Background

Insulin receptor (INSR) mutations, first described in 1988 [1, 2], are the most common cause of monogenic insulin resistance and hyperglycaemia. Homozygous INSR mutations are typically associated with two syndromes of severe insulin resistance; Donohue syndrome and Rabson-Mendenhall syndrome [3]. Both syndromes are characterised by intrauterine and postnatal growth restriction, facial dysmorphism and reduced life expectancy. Heterozygous mutations can cause type A insulin resistance (Type A IR), a less severe phenotype of IR, hyperandrogenism, oligomenorrhoea and acanthosis nigricans in the absence of obesity or lipoatrophy [3]. To date, only three cases of pregnant women with type A insulin resistance syndrome have been reported [4–6]. Small for gestational age (SGA) offspring have been reported in women with INSR mutations [5–7]. In this report, we present a woman with type A IR due to a heterozygous missense mutation in the β-subunit of the insulin receptor, p.Met1180Lys. We highlight phenotype variations, glycaemic management and therapeutic choice in pregnancy, neonatal outcomes and variable phenotypic expression in positive offspring.

## Case presentation

A 20 year old woman presented to the endocrine clinic in 2002 with secondary amenorrhoea, subfertility and hirsutism. Her father was known to have diabetes. On examination, blood pressure was 136/88 mmHg, her BMI was 28.6 kg/m^2^ and height 1.63 m with central adiposity and hirsutism. She had no clinical features of acanthosis nigricans, lipodystrophy or Cushing’s syndrome.

### Initial investigations

An oral glucose tolerance test (OGTT) revealed that she had diabetes mellitus with fasting glucose 12.3 mmol/l and 2 hour glucose 21.3 mmol/l and concurrent HbA1c 79 mmol/mol (9.4%). She was treated with metformin 500 mg twice daily. The ratio of luteinising hormone (14.3iu/l) to follicle stimulating hormone (5.9iu/l) was 2.4 and the free androgen index (FAI) was raised at 15 units (reference range 0.5–8.5). Overnight dexamethasone suppression testing ruled out Cushing’s syndrome (9 am cortisol < 50 nmol/l following 1 mg of dexamethasone). There was no evidence of polycystic ovaries on pelvic ultrasound.

### Follow up genetic diagnosis

The following year she attended our combined obstetric and diabetes clinic during her first pregnancy. Metformin was stopped in early pregnancy and she was started on insulin. She had one late pregnancy loss in 2003 and a preterm birth in 2005. Her first surviving child (offspring 2 in Table 1) was extremely preterm and SGA with a ventricular septal defect and pulmonary hypoplasia. She was diagnosed with hyperinsulinaemic hypoglycaemia at one year and managed with increased enteral feeds. At age nine years the child was diagnosed with a heterozygous missense INSR mutation of exon 20, p.Met1180Lys, c.3539 T > A, and this was then confirmed in her mother (age 32).

**Table 1:** Obstetric and neonatal characteristics of pregnancies extending beyond 20 weeks gestation

Offspring no.	1	2	3	4	5	6 (Twin 1)	7 (Twin 2)	8
**Maternal age**	21	22	33	34	35	36	36	38
**Gender**	M	F	F	M	F	F	M	M
**Genotype of offspring**	N/T	INSR+	INSR+	INSR+	INSR-	INSR+	N/T	N/T
Metformin	0	0	2 g/day	2 g/day	2 g/day	2 g/day until trimester 2	2 g/day until trimester 2	2 g/day
First trimester TDD insulin, units (unit/kg)	110 (1.47)	100 (1.27)	88 (1.25)	66 (0.91)	46 (0.66)	51 (0.68)	51 (0.68)	68 (0.9)
Third trimester TDD insulin, units (unit/kg)	120 (1.54)	134 (1.68)	83 (1.1)	71 (0.96)	46 (0.58)	111 (1.26)	111 (1.26)	120 (1.4)
Booking fructosamine (nmol/l)	280	283	304	365	333	331	331	368
Third trimester fructosamine (nmol/l)	–	–	230	235	224	224	224	235
Booking HbA1c (mmol/mol)	56	64	67	74	80	80	80	83
Third trimester HbA1c (mmol/mol)	–	–	39	45	55	55	55	52
Status at birth	Neonatal death	Live birth	Live birth	Live birth	Live birth	Live birth	IUD	IUD
Birthweight (kg)	0.5	0.74	2.87	3.11	3.52	2.2	2.5	3.52
Gestation (weeks)	21^+2^	26^+4^	38	38	38^+3^	36^+2^	36^+2^ (foetal demise at 32w)	35^+3^
Birth centile	n/a	5	40	66	94	9	100	100
Type of delivery	SVD	SVD	EmCS (failed IOL)	ELCS	ELCS	ELCS	ELCS	ELCS
Offspring complications	Placental insufficiency	HH, VSD, pulmonary hypoplasia, developmental delay, autism		T1DM age 2, IA-2, ZnT8 positive [8]				VSD

*M *male*, F *female*, N/T* not genetically tested, *INSR+* insulin receptor mutation positive, *INSR-* insulin receptor mutation negative, *TDD* total daily dose, *IUD* intrauterine death, *n/a* not applicable, *SVD* spontaneous vaginal delivery, *EmCS* emergency caesarean section, *IOL* induction of labour, *ELCS* elective caesarean section, *HH* hyperinsulinaemic hypoglycaemia, *VSD* ventricular septal defect, *T1DM* type 1 diabetes, *IA-2* islet tyrosine phosphatase 2 antibodies, *ZnT8* zinc transporter 8 antibodies

### Re-assessment following genetic diagnosis

On evaluation at age 37 in our centre in 2019, her BMI was 26.8 kg/m^2^. She underwent a repeat OGTT with insulin and C-peptide measurement shown in Table 2 consistent with severe insulin resistance (fasting insulin > 150 pmol/l). HbA1c was 97 mmol/mol (11.0%) as she was non-compliant with metformin and lifestyle measures. She had background diabetic retinopathy and urinary albumin creatinine ratio was normal at 1.7 g/mol. She has normal renal function (eGFR > 60 ml/min/1.73m^2^), ALT 20iu/l and GGT 18iu/l. Fasting lipid profile showed total cholesterol 5.6 mmol/l, raised triglycerides 3.91 mmol/l, LDL 3.5 mmol/l and HDL 1.18 mmol/l. Extended androgen profile was normal with 17-hydroxyprogesterone 4.4 nmol/l (< 5 nmol/l), androstenedione 2.2 nmol/l (1.0–9.6 nmol/l) and dehydroepiandrosterone sulfate 1.8umol/l (1.3–8.5umol/l). Leptin and adiponectin were normal at 16.3μg/l (8.6–38.9μg/l) and 5.0μg/ml (3.5–15.5μg/ml) respectively. Her glutamic acid decarboxylase (GAD) antibodies were positive at 1064 IU/ml in 2019 and 1247 IU/ml on repeat in 2023 (reference range 0-9iu/ml). Anti-islet cell and anti- insulin antibodies were negative.

**Table 2:** Glucose, insulin and C-peptide following 75gram OGTT

Time (min)	Glucose (mmol/l)	Insulin (pmol/l)	C-peptide (pmol/l)
−15	15.4	261.5	884
0	15.3	249.5	906
30	20.0	393.8	1259
60	24.9	505.1	1468
90	24.3	569.0	1833
120	23.4	544.2	1754

She was diagnosed with TSH receptor antibody positive Grave’s disease and treated with carbimazole 10 mg daily. Anti-tissue transglutaminase antibodies were positive and a duodenal biopsy confirmed villous atrophy consistent with Coeliac disease.

### Obstetric history

She had eleven pregnancies from a consanguineous relationship and smoked during all pregnancies. Eight pregnancies occurred after her genetic diagnosis. The outcomes of all pregnancies extending beyond 20 weeks gestation are shown in Table 1. All pregnancies were spontaneous bar the third pregnancy which required clomiphene therapy. Other pregnancies were unplanned and she was poorly compliant with metformin and folic acid prior to conception. She had five live births (offspring 2,3,4,5,6), one early neonatal death (offspring 1), two intrauterine deaths (offspring 7,8), three miscarriages and one ectopic pregnancy. Mean birthweight in INSR positive offspring (*n* = 3) was on the 37th centile (range 5-66th centile) excluding the twin pregnancy and intrauterine deaths.

**Fig. 1:**
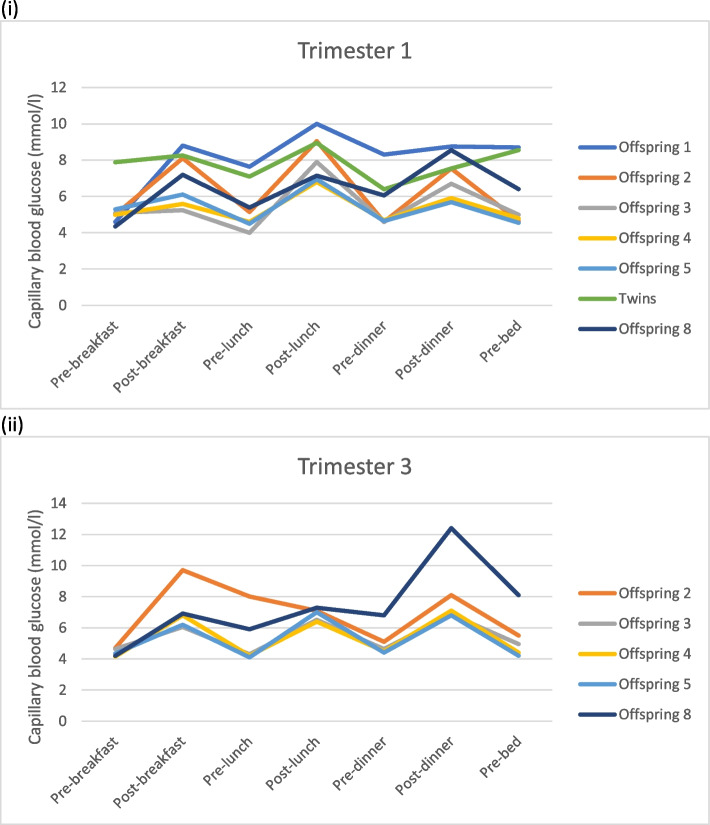
Mean self-monitored blood glucose profiles for each individual pregnancy for (**i**) one week in the first trimester (8 ± 4 weeks) and (**ii**) one week in the third trimester (29 ± 4.9 weeks)

At presentation to our combined diabetes and obstetric clinic she was commenced on insulin in all pregnancies. Optimal glycaemic control for pregnancy is defined by a corrected fructosamine value of less than 250 μmol/l. First trimester suboptimal control was seen in all pregnancies however optimal control was achieved by the second or third trimester. Glucose profiles from pregnancies are shown in Fig. 1. Target glucose levels were 3.5 mmol/l to 7.8 mmol/l. The maximum dose requirement of insulin was 134 units/day or 1.68 units/kg/day late in the second pregnancy. Metformin therapy was discontinued in the first two pregnancies, however metformin was used in all subsequent pregnancies. Insulin doses were lower in the metformin treated pregnancies (*n* = 5) vs insulin alone (*n* = 2) (mean first trimester dose 1.37 vs. 0.85unit/kg/day, and third trimester 1.61 vs. 1.1unit/kg/day).

### Neonatal outcomes

Foetal demise in the first pregnancy was attributed to placental insufficiency. The cases of intrauterine death involved suboptimal glycaemic control and large for gestational age offspring (100th centile following adjustment for gestational age) with a congenital ventricular septal defect evident on post-mortem in one case.

Five offspring (offspring 2,3,4,5,6 in Table 1) survived into childhood. Mean birthweight was in the 37th centile in INSR positive offspring from singleton pregnancies (*n* = 3) and 94th centile in the INSR negative offspring. INSR positive offspring 4 was diagnosed with autoimmune type 1 diabetes at age two having presented with diabetic ketoacidosis and IA-2A and ZnT8 antibody positivity [8]. Other siblings are clinically unaffected to date ranging in age from five to eight years old.

### Follow up & outcome

The patient is now age 41 and continues to attend our diabetes clinic. Compliance with medications is variable and HbA1c most recently was 99 mmol/mol. Fasting insulin and C-peptide are 321.6 pmol/l and 754 pmol/l at 21 years on from her diabetes diagnosis. Metformin has been escalated to maximum dose and pioglitazone introduced to increase insulin sensitivity. Carbimazole 5 mg once daily and a gluten free diet were prescribed. She has a Mirena coil in situ and does not plan any further pregnancies.

## Discussion

The prevalence of syndromes of insulin resistance is estimated to be in the order of 0.1–0.5% of all patients attending diabetes services [3]. Heterozygous INSR mutations, manifesting as type A IR, are under-recognised in clinical practice due to overlapping features of hyperandrogenism in women. Dominant heterozygous mutations involving the INSR β-subunit tyrosine kinase domain, p.Met1180Lys resulting in type A IR, has been previously described [6, 9].

Our case has some features of an INSR mutation; she has diabetes with hirsutism and oligomenorrhoea but no history suggestive of hyperinsulinaemic hypoglycaemia. Fasting insulin of 261.5 pmol/l indicates insulin resistance. Oligomenorrhoea and reduced fertility were prominent in early adulthood but appeared to regress with time as she achieved pregnancy spontaneously. The only other adult case of p.Met1180Lys described in the literature is a woman with a normal BMI who had diabetes in pregnancy only without clinical features of insulin resistance, hypoglycaemia or menstrual irregularities [6].

There are a number of aspects to this maternal case that are not common in INSR mutations. Raised triglycerides and low HDL are features of insulin resistance. The raised LDL in this case is not typically a feature of proximal insulin signaling disorders [10]. It more likely relates to suboptimal dietary measures.

The detection of GAD antibodies is unexpected in this case. The patient’s phenotype is not in keeping with type 1 diabetes mellitus as she is insulin resistant with a high c-peptide and insulin when measured 21 years following diabetes diagnosis and is not ketone prone. Notably, the subject has other autoimmune phenomena affecting her thyroid and Coeliac disease. Her son (offspring 4) also had antibody positive diabetes mellitus suggesting co-existence of an autoimmune trait in this family [8]. There are no reports of increased incidence of autoimmune disease in those with insulin receptor mutations and type A insulin resistance.

This is the first case report describing glucose management during pregnancy in a woman with a p.Met1180Lys mutation. We describe the medical and obstetric history based on the retrospective review of medical and obstetric case notes. Recurrent pregnancy loss is associated with insulin resistance [11], however the incidence of pregnancy loss in women with INSR mutations has not been established. The limitations of this report are that it describes a single maternal case. Multiple exposures in utero including the hyperglycaemia in early pregnancy and smoke exposure may have impacted on the low live birth rate of 42% (5/12 neonates) and increased risk of congenital anomalies.

1. Glycaemic control and insulin dose requirements in pregnancy

Insulin doses requirements in pregnancy were relatively in line with doses observed in women with type 2 diabetes. The maximum dose requirement of insulin was 1.68 units/kg/day comparable to doses in women with type 2 diabetes in the third trimester of pregnancy (up to 1.6 units/kg/day) [12]. To our knowledge, there is one other case in the literature describing insulin dose requirements in pregnancy complicated by type A IR as outlined in Table 3 [5]. Two offspring maternally inherited a heterozygous INSR deletion involving a loss of leucine in exon 17 (ΔLeu^999^). The first pregnancy was managed exclusively with insulin dose requirements up to 480 units/day in late pregnancy much higher than seen in our case. Combined use of metformin and insulin in a second pregnancy resulted in superior glycemic control and significantly reduced insulin dose requirements compared to the first pregnancy. These cases highlight the phenotypic variation that is seen with differing INSR mutations.

**Table 3:** INSR positive mutations, glycaemic control in pregnancy and birthweight

Reference	INSR location	Amino acid change	Age of presentation (years)	Maternal history of diabetes	Metformin in pregnancy, dose (g/day)	Third trimester insulin mean TDD (unit/kg)	Birthweight, gestation and centile, (kg and %)	Phenotype
Pedigree described in this case report	Exon 20	p.Met1180Lys	Early childhood	Yes	0	134 units/day	0.74, 26 weeks, 5th	HH
			Asymptomatic	Yes	2	83 units/day	2.87, 38 weeks, 40th	
			Asymptomatic	Yes	2	71 units/day	3.24, 38 weeks, 66th	T1DM [8]
			Asymptomatic	Yes	2^a^	111 units/day^a^	2.2, 36^+2^ weeks, twin	
Sethi et al. [6]	Exon 20	p.Met1180Lys	Newborn	Yes	No	Doses unknown	2.41, 37 weeks, 18th	HH
			Newborn	Yes	No	No	2.43, 38 weeks, 6th	HH
		p.Arg1110Gln	Newborn	No	–	–	2.025, 36 weeks, twin	HH
		p.Arg1191Gln	Newborn	No	–	–	2.2, 37 weeks, 5th	HH
Enkhtuvshin et al. [5]	Exon 17	ΔLeu^999^	Newborn	Yes	0	480 units/day	2.2, 37 weeks, 5th	Transient HH
			Newborn	Yes	2.5	150 units/day	2.5 kg, 38 weeks, 16th	Transient HH
Huang et al. [7]	Exon 20	p.Arg1174Trp	16	No	–	–	2.6 kg, 40 weeks, 5th	HH
			8	No	–	–	2.4 kg, twin	HH

*HH* hyperinsulinaemic hypoglycaemia, *T1DM* type 1 diabetes mellitus

^a^metformin therapy discontinued in late second trimester

This is the first case report to describe metformin use in pregnancy in this p.Met1180Lys INSR mutation. Metformin has been shown to be an effective and safe treatment option for gestational diabetes [13] however, it has been also associated with reduced neonatal birthweight and increases the risk of SGA infants [14]. One infant was SGA however metformin treatment was discontinued at diagnosis of pregnancy at five weeks gestation. Our experience suggests that metformin may be considered to improve insulin sensitivity in women with INSR mutations in pregnancy.

2. The role of INSR mutation positivity in birthweight

Maternal insulin resistance and elevated fasting glucose throughout pregnancy are associated with neonatal adiposity [15], independent of pre-pregnancy BMI and gestational weight gain [16]. INSR mutation positivity in offspring is associated with growth restriction [6]. A number of case reports have described maternal and neonatal outcomes in positive offspring as delineated in Table 3 [5–7]. The influence of maternal insulin resistance caused by INSR mutations on offspring without the mutation has not been described.

In our kindred, all pregnancies were insulin treated and associated with suboptimal glycaemic control in the first trimester impacting on final birthweight. One of the positive offspring was SGA (5th centile). The mean birthweight in genotype positive surviving offspring was on the 37th centile. The birthweight of the single genotype negative surviving offspring was relatively higher (94th centile).

One family with the same INSR mutation as our case was reported previously by Sethi et al. [6]. Two offspring inherited the p.Met1180Lys mutation. Both pregnancies were complicated by gestational diabetes with insulin treatment required in the latter stages of the first pregnancy only. The infant of the non-insulin treated pregnancy was SGA (6th centile), while the infant of the insulin treated pregnancy was not SGA (18th centile). Information on the degree of glycemic control and insulin dose requirements are not available for comparison although the degree of dysglycaemia was potentially less than our case as diabetes was identified in pregnancy only.

3. Incidence of hyperinsulinaemic hypoglycaemia

In the previously reported literature, two offspring inheriting the INSR mutation, p.Met1180Lys, both developed biochemically confirmed hyperinsulinaemic hypoglycaemia within 12 hours of birth and required diazoxide and chlorothiazide therapy until 8 and 11 months of age [6]. Within our pedigree, one of the four genetically confirmed carrier offspring manifested hyperinsulinaemic hypoglycaemia. This was diagnosed much later than the previous cases at one year old and was managed with increased frequency enteral feeding. The offspring who have inherited the missense INSR mutation without phenotypic features currently range in age from four to eight years old. This further supports the diversity in the clinical phenotype of INSR mutations [17, 18].

## Conclusion

INSR mutations are rare. The p.Met1180Lys mutation results in a phenotype of diabetes, hirsutism and oligomenorrhoea. This woman had co-existence of autoimmune disease. Her insulin dose requirements during pregnancy were similar to doses observed in women with type 2 diabetes. Metformin may be used to improve insulin sensitivity in women with INSR mutations in pregnancy. Offspring inheriting the mutation tended to be smaller for gestational age. In future, pre-pregnancy diagnosis of INSR in women may help clarify optimal management of both women and offspring.

## Data Availability

Data to support the findings in this case report can be requested from the corresponding author.

## Abbreviations

INSR: insulin receptor
TypeA IR: type-A insulin resistance
IR: insulin resistance
SGA: small for gestational age
BMI: body mass index
LH: luteinising hormone
FSH: follicle stimulating hormone
FAI: free androgen index
OGTT: oral glucose tolerance test
ALT: alanine transaminase
GGT: gamma-glutamyl transpeptidase
LDL: low-density lipoprotein
HDL: high-density lipoprotein
GAD: glutamic acid decarboxylase
N/T: not genetically tested
INSR+: insulin receptor mutation detected
INSR-: insulin receptor mutation not detected
TDD: total daily dose
IUD: intra-uterine death
SVD: spontaneous vaginal delivery
EmCS: emergency Caesarean section
ELCS: elective Caesarean section
HH: hyperinsulinaemic hypoglycaemia
VSD: ventricular septal defect
T1DM: type 1 diabetes mellitus
IA-2: islet tyrosine phosphatase 2
ZnT8: zinc transporter 8
